# Supplementary material for: From the dual cyclone harvest performance of single conidium powder to the effect of Metarhizium anisopliae on the management of Thaumastocoris peregrinus (Hemiptera: Thaumastocoridae)
Source: PLoS One. 2023 Mar 27;18(3):e0283543. doi: 10.1371/journal.pone.0283543 (PMC10042367; doi:10.1371/journal.pone.0283543)
Supplement: Attachment — Submitted filename: PONE-D-22-33367.pdf [file pone.0283543.s001.pdf]

PLOS ONE

From the dual cyclone harvest performance of single conidium powder to the effect of Metarhizium anisopliae on the management of a forest pest

--Manuscript Draft--

**Manuscript Number:**	PONE-D-22-33367
**Article Type:**	Research Article
**Full Title:**	From the dual cyclone harvest performance of single conidium powder to the effect of Metarhizium anisopliae on the management of a forest pest
**Short Title:**	Dual cyclone harvest performance and effect of Metarhizium anisopliae on a forest pest
**Corresponding Author:**	Maurício Magalhães Domingues<br>Universidade Estadual Paulista Júlio de Mesquita Filho Câmpus de Botucatu<br>Faculdade de Ciências Agronômicas<br>Botucatu, SP BRAZIL
**Keywords:**	bronze bug; entomopathogenic fungus; mycoinseticides; Mycoharvester®; pure conidia
**Abstract:**	Insect pests introduced to eucalyptus plantations in Brazil are mostly of Australian origin, but the potential of their management using native microorganisms is high. The quality of biopesticide production based on entomopathogenic fungi depends on adequate technology. The objective of this study was to evaluate Mycoharvester® equipment for harvesting and separating particles to obtain pure Metarhizium anisopliae conidia to manage Thaumastocoris peregrinus Carpintero & Dellapé, 2006 (Hemiptera: Thaumastocoridae). The Mycoharvester® version 5b harvested and separated M. anisopliae spores. The pure conidia were suspended in Tween 80® (0.1%) and calibrated to concentrations of 1 x 106, 107, 108 and 109 conidia/ml to evaluate fungus pathogenicity, lethal concentration 50 and 90 (LC50, LC90) and lethal time 50 and 90 (LT50, LT90) to T. peregrinus. This equipment harvested 85% of the conidia from rice, with production of 4.8 ± 0.38 x 109 conidia/g dry mass of substrate + fungus. The water content (6.36%) of the single spore powder (pure conidia) separated by the Mycoharvester® compared to the moisture of the agglomerated product was low. The product harvested caused high mortality of T. peregrinus nymphs and adults. The separation of conidia produced by solid-state fermentation with the Mycoharvester® is an important step toward optimizing the fungal production system of pure conidia, and to formulating biopesticides for insect pest management.
**Order of Authors:**	Simone Graziele Moio Velozo
	Murilo Rodrigues Velozo
	Maurício Magalhães Domingues
	Luciane Katarine Becchi
	Vanessa Rafaela de Carvalho
	José Raimundo de Souza Passos
	José Cola Zanuncio
	José Eduardo Serrão
	Dietrich Stephan
	Carlos Frederico Wilcken
**Additional Information:**	
**Question**	**Response**
**Financial Disclosure**<br><br>Enter a financial disclosure statement that	Funding was provided by the following Brazilian institutions: “Conselho Nacional de Desenvolvimento Científico e Tecnológico (CNPq)”, Coordenação de Aperfeiçoamento de Pessoal de Nível Superior – Brazil (CAPES) granted the the scholarship in

Powered by Editorial Manager® and ProduXion Manager® from Aries Systems Corporation

describes the sources of funding for the work included in this submission. Review the submission guidelines for detailed requirements. View published research articles from *PLOS ONE* for specific examples.<br><br>This statement is required for submission and **will appear in the published article** if the submission is accepted. Please make sure it is accurate.<br><br>**Unfunded studies**<br>Enter: *The author(s) received no specific funding for this work.*<br><br>**Funded studies**<br>Enter a statement with the following details:<br>• Initials of the authors who received each award<br>• Grant numbers awarded to each author<br>• The full name of each funder<br>• URL of each funder website<br>• Did the sponsors or funders play any role in the study design, data collection and analysis, decision to publish, or preparation of the manuscript?<br>• **NO** - Include this sentence at the end of your statement: *The funders had no role in study design, data collection and analysis, decision to publish, or preparation of the manuscript.*<br>• **YES** - Specify the role(s) played.<br><br>* typeset	Germany (Financing Code 88881.134760/2016-01) and in Brazil (Financing Code 001) to S.G.M.V. and Programa Cooperativo sobre Proteção Florestal/PROTEF do Instituto de Pesquisas e Estudos Florestais/IPEF.
**Competing Interests**<br><br>Use the instructions below to enter a competing interest statement for this submission. On behalf of all authors, disclose any competing interests that could be perceived to bias this work—acknowledging all financial support and any other relevant financial or non-financial competing interests.<br><br>This statement is required for submission and **will appear in the published article** if the submission is accepted. Please make sure it is accurate and that any funding	The authors have declared that no competing interests exist.

sources listed in your Funding Information later in the submission form are also declared in your Financial Disclosure statement.<br><br>View published research articles from *PLOS ONE* for specific examples.<br><br>**NO authors have competing interests**<br><br>Enter: *The authors have declared that no competing interests exist.*<br><br>**Authors with competing interests**<br><br>Enter competing interest details beginning with this statement:<br><br>*I have read the journal's policy and the authors of this manuscript have the following competing interests: [insert competing interests here]*<br><br>* typeset	
**Ethics Statement**<br><br>Enter an ethics statement for this submission. This statement is required if the study involved:<br><br>• Human participants<br>• Human specimens or tissue<br>• Vertebrate animals or cephalopods<br>• Vertebrate embryos or tissues<br>• Field research<br><br>Write "N/A" if the submission does not require an ethics statement.<br><br>General guidance is provided below. Consult the submission guidelines for detailed instructions. **Make sure that all information entered here is included in the Methods section of the manuscript.**	No specific permits are required to rear T. peregrinus in Brazil. The services for fungi production and harvest were executed in Germany, by a legal document pursuant to § 6 Article 24 of Decree No. 8.772, dated May 11th, 2016, which regulates National Law No. 13,123, of May 20th, 2015, providing access to genetic heritage, protection and access to associated traditional knowledge and the sharing of benefits for the conservation and sustainable use of biodiversity in Brazil. This was signed between the UNESP/FCA and the Julius Kühn Institute. The registration of the project was carried out in SISGEN. The isolate IBCB425 was returned to Brazil at the end of this research. The laboratory studies did not involve endangered or protected species in either Brazil and Germany.

Powered by Editorial Manager® and ProduXion Manager® from Aries Systems Corporation

**Format for specific study types**<br><br>**Human Subject Research (involving human participants and/or tissue)**<br>• Give the name of the institutional review board or ethics committee that approved the study<br>• Include the approval number and/or a statement indicating approval of this research<br>• Indicate the form of consent obtained (written/oral) or the reason that consent was not obtained (e.g. the data were analyzed anonymously)<br><br>**Animal Research (involving vertebrate animals, embryos or tissues)**<br>• Provide the name of the Institutional Animal Care and Use Committee (IACUC) or other relevant ethics board that reviewed the study protocol, and indicate whether they approved this research or granted a formal waiver of ethical approval<br>• Include an approval number if one was obtained<br>• If the study involved *non-human primates*, add *additional details* about animal welfare and steps taken to ameliorate suffering<br>• If anesthesia, euthanasia, or any kind of animal sacrifice is part of the study, include briefly which substances and/or methods were applied<br><br>**Field Research**<br><br>Include the following details if this study involves the collection of plant, animal, or other materials from a natural setting:<br>• Field permit number<br>• Name of the institution or relevant body that granted permission	
**Data Availability**<br><br>Authors are required to make all data underlying the findings described fully available, without restriction, and from the time of publication. PLOS allows rare exceptions to address legal and ethical concerns. See the PLOS Data Policy and FAQ for detailed information.	Yes - all data are fully available without restriction

Powered by Editorial Manager® and ProduXion Manager® from Aries Systems Corporation

A Data Availability Statement describing where the data can be found is required at submission. Your answers to this question constitute the Data Availability Statement and **will be published in the article**, if accepted.<br><br>**Important:** Stating 'data available on request from the author' is not sufficient. If your data are only available upon request, select 'No' for the first question and explain your exceptional situation in the text box.<br><br>Do the authors confirm that all data underlying the findings described in their manuscript are fully available without restriction?	
**Describe where the data may be found in full sentences. If you are copying our sample text, replace any instances of XXX with the appropriate details.**<br><br>• If the data are **held or will be held in a public repository**, include URLs, accession numbers or DOIs. If this information will only be available after acceptance, indicate this by ticking the box below. For example: *All XXX files are available from the XXX database (accession number(s) XXX, XXX.*).<br>• If the data are all contained **within the manuscript and/or Supporting Information files**, enter the following: *All relevant data are within the manuscript and its Supporting Information files.*<br>• If neither of these applies but you are able to provide **details of access elsewhere**, with or without limitations, please do so. For example:<br><br>*Data cannot be shared publicly because of [XXX]. Data are available from the XXX Institutional Data Access / Ethics Committee (contact via XXX) for researchers who meet the criteria for access to confidential data.*<br><br>*The data underlying the results presented in the study are available from (include the name of the third party*	All data generated or analysed during this study are included in this manuscript.

and contact information or URL).<br>• This text is appropriate if the data are owned by a third party and authors do not have permission to share the data.<br><br>* typeset	
Additional data availability information:	

Manuscript

Click here to access/download;Manuscript;12.02.22.PLOSone.dual_cyclone

PLOS ONE

From the dual cyclone harvest performance of single conidium powder to the effect of Metarhizium anisopliae on the management of a forest pest

Simone Graziele Moio Velozo[1,5], Murilo Rodrigues Velozo[2], Maurício Magalhães Domingues[1*], Luciane Katarine Becchi[1], Vanessa Rafaela de Carvalho[1], José Raimundo de Souza Passos[3], José Cola Zanuncio[4], Dietrich Stephan[5] and Carlos Frederico Wilcken[1]

[1] Laboratório de Controle Biológico de Pragas Florestais, Faculdade de Ciências Agronômicas, Universidade Estadual Paulista (UNESP), 18610-034, Botucatu, São Paulo, Brasil

[2] Syntech Research, 13400-430, Piracicaba, São Paulo, Brasil

[3] Departamento de Bioestatística, Instituto de Biociências, Universidade Estadual Paulista (UNESP), 18618-689, Botucatu, São Paulo, Brasil

[4] Departamento de Entomologia/BIOAGRO, Universidade Federal de Viçosa, 36570-900, Viçosa, Minas Gerais, Brasil

[5] Julius Kühn-Institut, Federal Research Centre for Cultivated Plants, Institute for Biological Control, 64287 Darmstadt, Germany

* Corresponding author

E-mail: mauricio.m.domingues@unesp.br

Abstract

Insect pests introduced in eucalyptus plantations in Brazil are mostly of Australian origin, but native microorganisms have potential for their management. High quality biopesticide production based on entomopathogenic fungi depends on adequate technology. The objective of this study was to evaluate Mycoharvester® equipment for harvesting and separating particles to obtain pure *Metarhizium anisopliae* conidia to manage *Thaumastocoris peregrinus* Carpintero & Dellapé, 2006 (Hemiptera: Thaumastocoridae). The Mycoharvester® version 5b harvested and separated *M. anisopliae* spores. The pure conidia were suspended in Tween 80® (0.1%) and calibrated to concentrations of 1 x 10^6, 10^7, 10^8 and 10^9 conidia/ml to evaluate fungus pathogenicity, lethal concentration 50 and 90 (LC_{50}, LC_{90}) and lethal time 50 and 90 (LT_{50}, LT_{90}) to *T. peregrinus*. This equipment harvested 85% of the conidia from rice, with production of 4.8 ± 0.38 x 10^9 conidia/g dry mass of substrate + fungus. The water content of 6.36% of the single spore powder (pure conidia) separated by the Mycoharvester® shows lower water content compared to the moisture of the agglomerated product. The product harvested caused high *T. peregrinus* nymph and adult mortality. The separation of conidia produced by solid-state fermentation with the Mycoharvester® is an important step toward optimizing the fungal production system of pure conidia, and to formulating biopesticides for insect pest management.

Author Summary

Our work is a valuable addition to the scientific literature because we clarified the following hypotheses: (a) harvesting technology (Mycoharvester) can be applied for fungal Brazilian isolate (IBCB425), (b) the pure conidia obtained by this technology is effective to manage *T. peregrinus*. Entomopathogenic fungi can reduce arthropod-pest

infestations when used as biopesticide. The objective was to evaluate the *M. anisopliae* harvest from the substrate using an automated method with a dual cyclone technology, which collects and separates particles, aiming the obtaining of pure conidia. Also testing the pure conidia to manage *T. peregrinus* (nymph and adult) under laboratory conditions. The production and use of entomopathogenic fungi have grown worldwide. However, few countries have a high technological level in this sector. Developing countries depend on intensive labor methods for the production of fungi. The isolate IBCB425 is already registered in the Ministry of Agriculture in Brazil and is active ingredient of many formulations by several companies. However, the harvest of *M. anisopliae* (IBCB425) using Mycoharvester and the use of this fungus to control *T. peregrinus* was not known before this present research.

Introduction

Planted forests contribute to the Brazilian economy with 7.8 million hectares, of which 72% are *Eucalyptus* spp., providing 91% of the wood produced in the country [1]. Exotic insect pests such as *Thaumastocoris peregrinus* Carpintero & Dellapé, 2006 (Hemiptera: Thaumastocoridae), *Glycaspis brimblecombei* Moore, 1964 (Hemiptera: Aphalaridae) and *Gonipterus platensis* Marelli, 1926 (Coleoptera: Curculionidae) threaten these numbers [2,3,4]. *Thaumastocoris peregrinus*, a pest in *Eucalyptus* spp. plantations, has a high dispersion capacity and damages different species of this genus [5,6].

Monitoring is essential to determine *T. peregrinus* adult, nymph and egg numbers in the plant canopy, to assist in managing this pest [7]. The biological control of *T. peregrinus* using the parasitoid *Cleruchoides noackae* Lin & Huber, 2007 (Hymenoptera: Mymaridae) [7], insect predators [8,9] and entomopathogenic fungi, is important to manage this and other forest insects [10,11,12]. However, the production of these

microorganisms in developing countries requires intense manual labor, which can reduce the quantity and quality of the final product [13,14]. This increases the difficulties of obtaining large quantities of biopesticides of acceptable quality to meet the requirements of forest and agricultural areas.

A total of 171 mycoinsecticides were produced with at least 12 of the more than 800 species of entomopathogenic fungi, emphasizing the genera *Beauveria*, *Cordyceps* and *Metarhizium*, with the formulations of technical concentrates in the form of substrates colonized by fungi, wettable powders and dispersions in oil being the most common [15]. The dual cyclone technology is a method used in conidia harvesting, with advantages in the elimination of large particles, a high-quality spore separation, operator safety, economical convenience processing, ease of storage and higher conidia productivity, such as for *Beauveria bassiana* and *Metarhizium anisopliae* [16]. This technology must be effectively explored with a view to its use in the formulation and application of mycoinsecticides, contributing directly to the integrated management of agriculture and forest pests. The objective of this study is to evaluate harvesting technology with particle separation to obtain pure conidia of *Metarhizium anisopliae* (IBCB425), and to evaluate the pathogenicity of the product obtained against *T. peregrinus*.

Results

Molecular analysis

The sequence obtained in the molecular analysis showed coverage and high identity, with 100% for the species *Metarhizium anisopliae* (GenBank accession number MT278261.1).

Product harvest and the Mycoharvester® system

The *M. anisopliae* yield was 4.8 ± 0.38 x 10^9 conidia/g of dry mass of substrate + fungus with water content of 57.7 ± 1.06%. A total of 150 g of substrate colonized by 4.8 ± 0.38 x 10^9 conidia/g of the fungus provided 3.12 ± 0.21 g of pure conidia with 5.31 ± 0.28 x 10^{10} conidia/g, and 1.14 ± 0.09 g of the agglomerate with 1.16 ± 0.24 x 10^{10} conidia/g (Fig 1a). The word “agglomerate” means a product with conidia, mycelia and residues from the substrate. The substrate + fungus yield in the Mycoharvester® version 5b (Fig 2a) before and after (Figs 1b, 2b and 2c) using this system was 4.8 ± 0.38 x 10^9 conidia/g and 6.9 ± 0.71 x 10^8 conidia/g dry mass, respectively, confirming that this method collects approximately 85% of the conidia, where approximately 73% is pure conidia (Figs 1a and 2d). The water content of the products differed, with 6.36 ± 0.13% for pure conidia and 34.84 ± 0.65% for the agglomerate (Fig 1c).

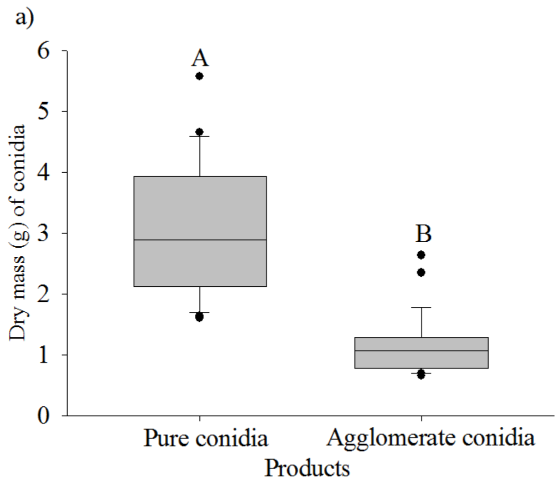

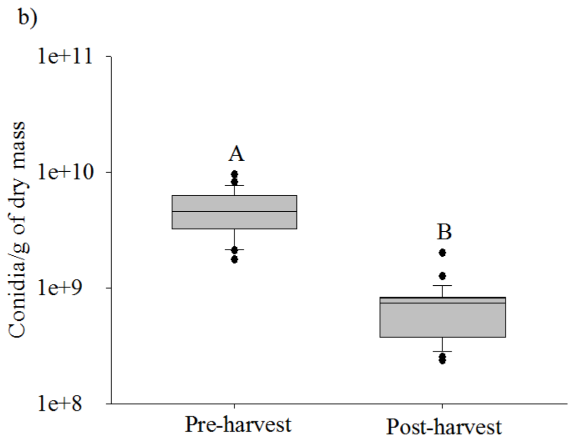

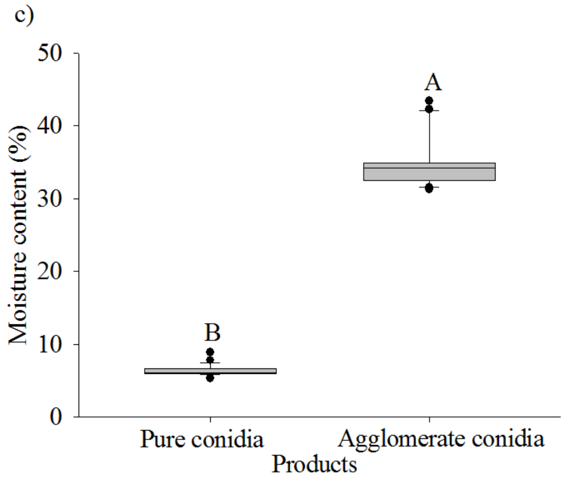

Fig 1. Evaluation of conidia harvest with Mycoharvester® System. a: Dry mass (g) of the pure and agglomerate conidia in 150g of colonized substrate (P< 0.0001). b: water content of the pure and agglomerate conidia of *Metarhizium anisopliae* after harvesting of material (P < 0.0001). c: performance of Mycoharvester® version 5b in the conidia harvest of *Metarhizium anisopliae*: comparison of the substrate + fungus before and after the harvest procedure (P< 0.0001). Means followed by different capital letters differ by the Tukey-Kramer test at 5% significance level.

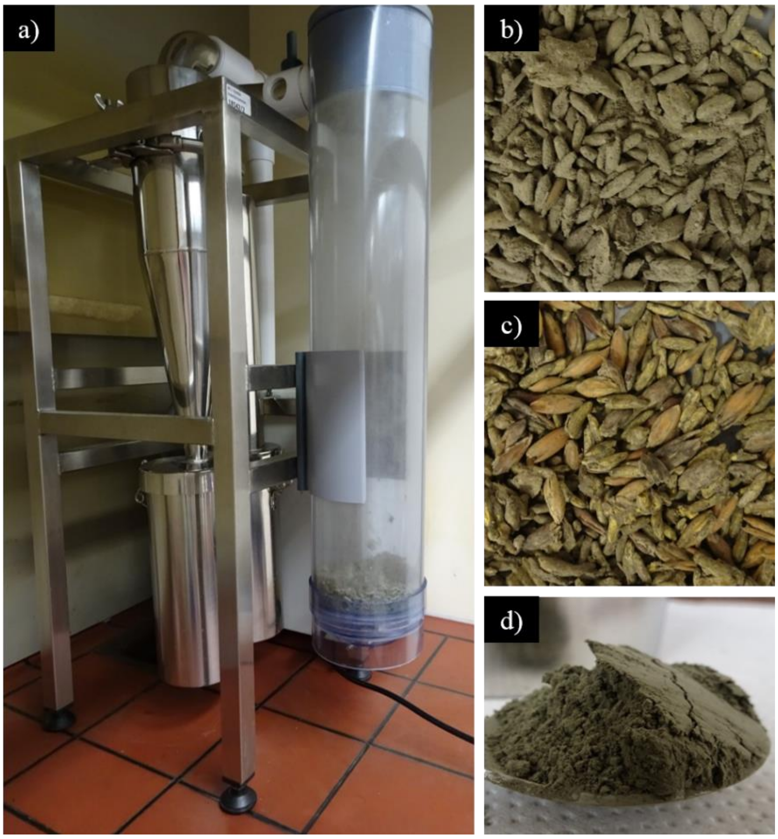

Fig 2. Mycoharvester® System. Mycoharvester® equipment, version 5b (a); substrate colonized by *Metarhizium anisopliae* before (b) and after harvest (c); pure conidia of *Metarhizium anisopliae* (d).

Pathogenicity to *Thaumastocoris peregrinus*

The pure conidia of *M. anisopliae* (IBCB425), applied at different concentrations, was pathogenic to *T. peregrinus* nymphs and adults, being more virulent at concentrations of 10^8 and 10^9 conidia/ml ($P < 0.05$). All treatment averages differed from that of the control ($P < 0.05$), except the 10^6 conidia/ml concentration applied to nymphs and to nymphs and adults together (Fig 3 and Table 1).

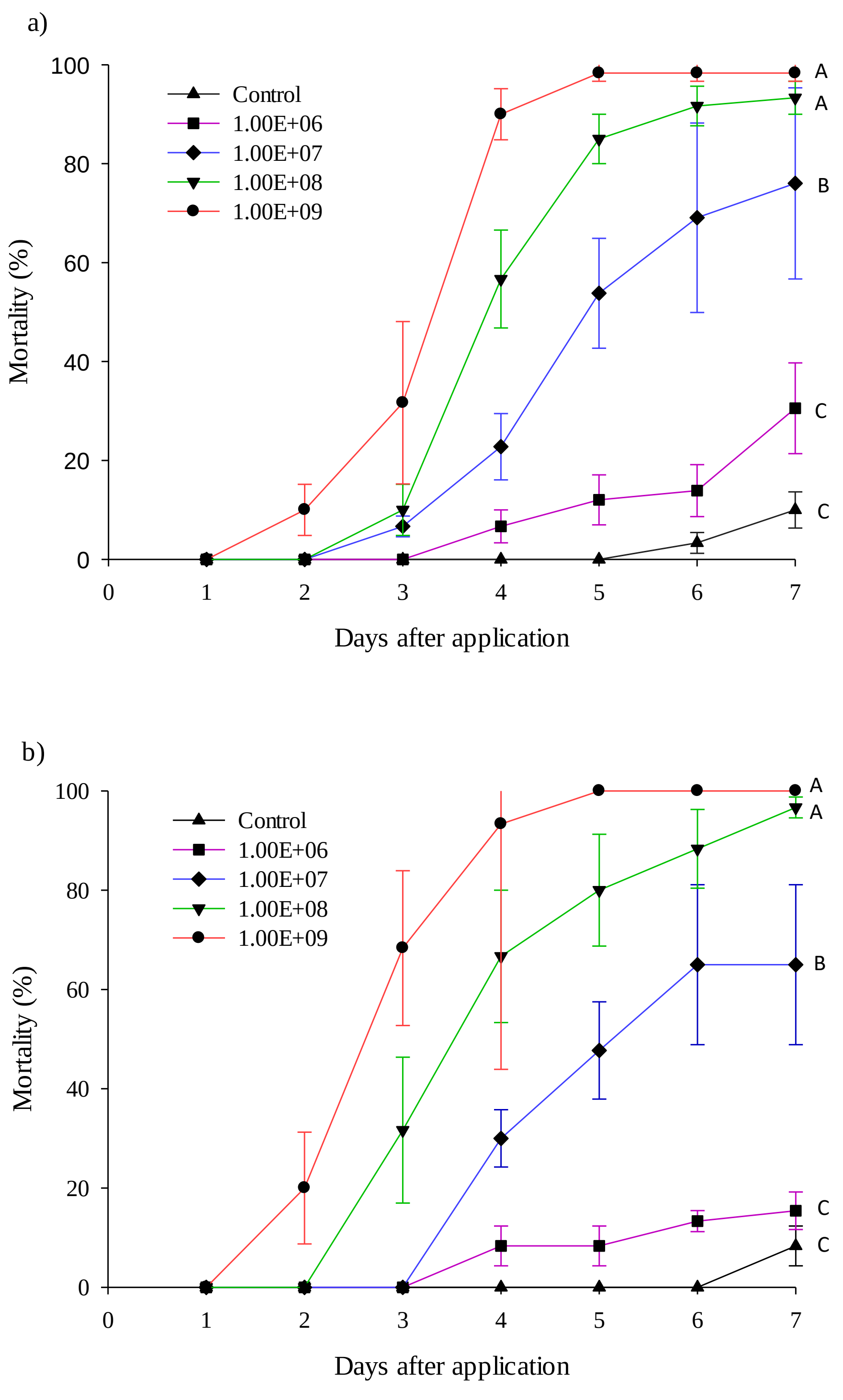

Fig 3. Cumulative pathogenicity of *Metarhizium anisopliae* in different concentrations of pure conidia to adults (a) and nymphs (b) of *Thaumastocoris peregrinus* (Hemiptera: Thaumastocoridae) after seven days of application. Means followed by different capital letters differ by the Tukey-Kramer test at the 5% significance level.

Table 1. Mortality of nymphs and adults of *Thaumastocoris peregrinus* (Hemiptera: Thaumastocoridae) with *Metarhizium anisopliae* (IBCB425) (%) in pure conidia concentrations (conidia/mL)

IBCB425	Nymph	Adult	Total
Control	10.00 ± 3.65 C	8.33 ± 4.01 C	9.17 ± 2.60 C
10^6	28.33 ± 8.33 B	15.00 ± 3.42 C	21.67 ± 4.74 C
10^7	55.00 ± 8.85 B	50.00 ± 10.00 B	52.50 ± 6.41 B
10^8	93.33 ± 3.33 A	96.67 ± 2.11 A	95.0 ± 1.95 A
10^9	98.33 ± 1.67 A	100.00 ± 0.00 A	99.17 ± 0.83 A

Means followed by the same capital letter per column do not differ by the Tukey-Kramer test (Westfall, et al., 1999) at 5% significance.

The *T. peregrinus* nymphs and adults killed by *M. anisopliae* (IBCB425) presented conidiogenesis, and it was possible to observe the fungus sporulation on their body (Fig 4).

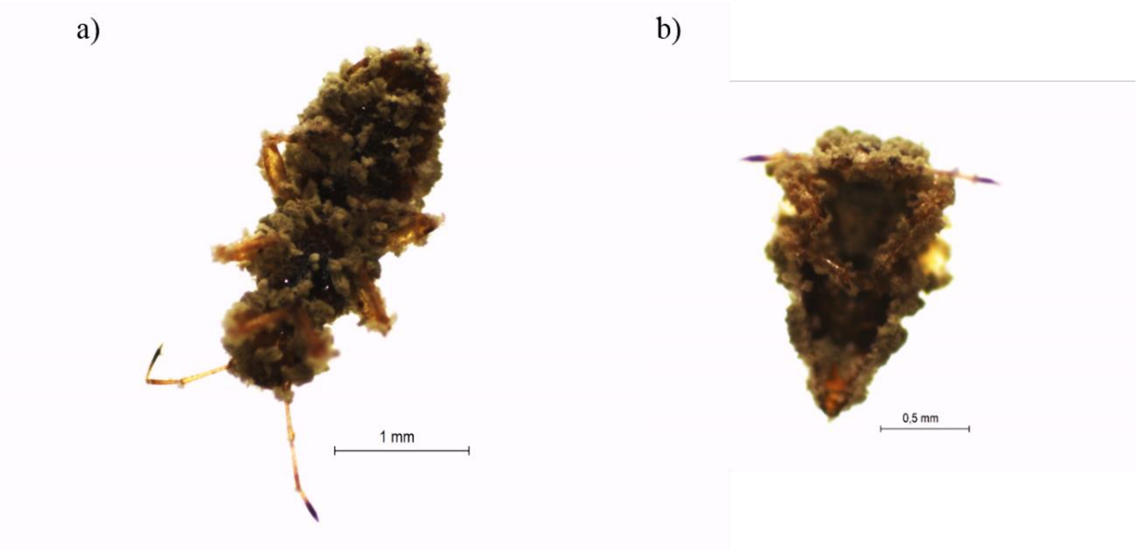

Fig 4. Adult (a) and nymph (b) of *Thaumastocoris peregrinus* (Hemiptera: Thaumastocoridae) colonized by *Metarhizium anisopliae*.

The IBCB425 isolate presented lower 50 and 90 lethal times with the higher concentrations 10^8 and 10^9 conidia/ml, needing 4.85 (4.53 – 5.28) and 3.22 (3.04 – 3.43) days and 7.37 (6.69 – 8.37) and 4.61 (4.27 – 5.09) days, respectively, to kill 50% and 90% of the *T. peregrinus* population (Table 2). The lethal concentration to kill 50 of the population was, respectively, 6.4 x 10^7 (2.7 x 10^6 – 1.6 x 10^7) and 1.5 x 10^9 (5.5 x 10^8 – 5 x 10^9) conidia/ml.

Table 2. Lethal time (50 and 90) of *Metarhizium anisopliae* (IBCB425) (days) at different concentrations (conidia/mL) in the mortality of *Thaumastocoris peregrinus* nymphs and adults (Hemiptera: Thaumastocoridae)

IBCB425	Lethal time 50	Lethal time 90
Control	8.27 (7.63 – 11.30)	9.40 (8.33 – 14.73)
10^6	11.42 (9.38 – 17.14)	15.71 (12.31 – 25.48)
10^7	10.65 (8.75 – 15.06)	16.97 (13.28 – 25.75)
10^8	4.85 (4.53 – 5.28)	7.37 (6.69 – 8.37)
10^9	3.22 (3.04 – 3.43)	4.61 (4.27 – 5.09)

Discussion

The molecular analysis confirmed that IBCB425 has been reported and recorded in Brazil, as *M. anisopliae* [26,27,28].

Production in a solid bioreactor was adequate to generate significant quantities of pure conidia of *M. anisopliae* (IBCB425). The production of this fungus in solid-state fermentation with rice as a substrate is around 10^9 conidia/g, although the optimization of factors such as substrate moisture, between 22 and 30% of rice moisture, can increase these values, demonstrating the efficiency of this method [29,30]. The quality of *M.*

anisopliae production in a solid-state bioreactor with 3.4 x 10^9 conidia/g obtained with rice bran was the best in a test with different substrates in the aerated packed beds [31].

The high yield of conidia collection and separation in the Mycoharvester® is similar to that reported for this technique with *B. bassiana* and *M. anisopliae*, with 12.6 mg/g and 1.8×10^{10} conidia/g, presenting yield superior to that from using sieving and washing methods with Tween 80 (0.02%) [16]. Aerial conidia is the principal and most effective infectious stage of the entomopathogenic Hypocreales [32] and the ease of production of pure conidium in the technique studied indicates its potential for large-scale production and application. Mycoharvester® improved the harvesting of pure conidia without using water, which may impair the stability and purity of the product, to develop new oily formulations based on *Metarhizium* spp. to control locusts in Africa [33].

The water content of the products, 6% for pure conidia and 34% for the agglomerate, shows the adequacy of the Mycoharvester® for conidia separation in developing biopesticides based on entomopathogenic fungi. The water content of conidia from solid substrates must be lower than 9% to allow a better shelf life, regardless of whether the product is formulated [34,32,13]. The water content of the conidia affects its germination capacity during storage periods, and relative humidity of 2.5% to 6.2% was ideal for the high germination of *Metarhizium flavoviride* conidia, even after being exposed to conditions of 50°C [35]. However, dry infective propagules, when immersed in water for rehydration, can severely damage plasma membranes, which is usually lethal in single-celled organisms [36]. This occurred for dried conidia of *M. anisopliae* suspended in water at 0.5°C and 33°C, with germination rates of 0.9% and 94%, respectively, when rehydrated, showing the importance of care during rehydration [36].

The high *T. peregrinus* mortality with *Metarhizium anisopliae* (IBCB425) indicates its potential for managing this pest with its conidia applied in suspension, as reported for

different *M. anisopliae* isolates, including IBCB425 and IBCB348, with mortality of up to 100% of other Hemiptera, such as the sugarcane spittlebug *Mahanarva fimbriolata* Stål (Hemiptera: Cercopidae) [27], and adults of *Diaphorina citri* Kuwayama, 1908 (Hemiptera: Liviidae) in the laboratory [37]. Native entomopathogenic fungi can improve control of exotic forest pests in sustainable management programs, as with successful cases of efficiency or natural occurrence of fungi such as *B. bassiana*, *Cordyceps* sp. and *Zoophtora radicans* [11,38,39].

The isolate IBCB425 was more virulent to *T. peregrinus* at the highest concentrations, requiring 6.4 x 10^7 (2.7 x 10^7 – 1.6 x 10^8) and 1.5 x 10^9 (5.5 x 10^8 – 5 x 10^9) conidia/ml to control 50 and 90%, respectively, of insects in both stages (nymph and adult), which means 10^{11} to 10^{13} conidia/ha. These values make it feasible to use entomopathogenic fungi in field conditions for *T. peregrinus*, since commercial products are generally commercialized and recommended at the field concentration of 1 x 10^{12} to 1 x 10^{13} conidia/ha [40,41,42]. The concentration of 5 x 10^{12} conidia/ha controlled *D. citri* up to 95.6% (*B. bassiana*) and 94% (*Cordyceps fumosorosea*) under field conditions [42]. Commercial products based on *B. bassiana* and *M. anisopliae* at a concentration of 1 x 10^8 conidia/ml caused 100 and 88% mortality of *T. peregrinus* adults, respectively, after 11 days under laboratory conditions [43], the same as the commercial isolate tested in our bioassays. The LC_{50} of *B. bassiana* blastospores, produced in a submerged medium, was higher than observed for IBCB425, approximately 3 x 10^7 conidia/ml for three different isolates, while it did not differ from the LC_{50} of aerial conidia [44]. Different isolates of *M. anisopliae*, at concentrations of 10^5, 10^6, 10^7 and 10^8, were pathogenic at the highest concentrations to *Rhipicephalus* (*Boophilus*) *microplus* Canestrini, 1888 (Acari: Ixodida) [45]. The concentration of 1.2 x 10^7 conidia/ml of *M. anisopliae* (IBCB348) caused higher *M. fimbriolata* mortality [46]. Formulations based on *B. bassiana*, *M. anisopliae* and

Lecanicillium lecanii were pathogenic to *G. brimblecombei*, with increased mortality as the conidia concentration increased [10].

The use of native entomopathogenic fungi to manage introduced forest pests increases the viability of sustainability programs. Naturally occurring entomopathogenic fungi such as *B. bassiana*, *Cordyceps* spp., *Beauveria pseudobassiana* and *Zoophtora radicans* are common in the field, and these species show promise against *T. peregrinus* [38,39,47]. *T. peregrinus* mortality, from 80.1% to 100% with *B. bassiana*, 88% with *M. anisopliae*, and 87% with *Cordyceps* sp. at a concentration of 10^8 conidia/ml, and 98% with *B. bassiana* at 10^7 conidia/ml, which demonstrates the potential of these microorganisms to manage this pest [12,43,47]. Successful cases in the agricultural sector with entomopathogenic fungi have been reported, especially with *M. anisopliae*, for decades, against Hemiptera pests in sugarcane cultures in Brazil [14]. The high pathogenicity of the final product obtained with the dual cyclone harvest technique using Mycoharvester, with results similar to formulated products efficient against insect pests, offers significant efficiency for the collection and production of pure conidia without interfering with the action of entomopathogenic fungi. This is important to improve and develop mycoinsecticides and highlights the relevance of this still poorly investigated technology.

The Mycoharvester® equipment is highly efficient for the collection, separation, and quality of pure *M. anisopliae* conidia. The pure conidia obtained with this technology shows potential to manage *T. peregrinus* adults and nymphs.

Materials and Methods

Obtaining the fungus by solid fermentation

The fungus *Metarhizium anisopliae sensu lato* (Hypocreales: Clavicipitaceae) (IBCB425) was isolated from Atlantic Forest soil collected in Iporanga, São Paulo,

Brazil, and deposited in the Oldemar Cardim Abreu Entomopathogenic Microorganism Collection at the "Centro Avançado de Pesquisa em Proteção de Plantas e Saúde Animal" (Advanced Center for Research into the Protection of Plant and Animal Health) in Campinas, São Paulo, Brazil. Large quantities of IBCB425 (strain) conidia were obtained with a solid-state fermentation process at the Julius Kühn-Institute, Darmstadt, Germany. The ProPhyta L03 Fermenter System® [Prophyta, Germany] laboratory bioreactor was filled with a substrate of parboiled rice[a] + barley[b], 4:1 (160g[a] + 40g[b]) to mass-produce this fungus at 25°C with an aeration rate of 150 L/h [17]. The *M. anisopliae* conidia were harvested after 14 days of fermentation and one day for extraction and quantification.

Obtaining *Thaumastocoris peregrinus*

Branches infested with *Thaumastocoris peregrinus* adults were collected from *Eucalyptus grandis* × *E. urophylla* hybrid plants and taken for mass rearing at the Laboratory of Biological Control of Forest Pests (LCBPF), of the Department of Plant Protection, Faculdade de Ciências Agronômicas, Universidade Estadual Paulista Júlio de Mesquita Filho - FCA, UNESP (College of Agronomic Sciences, São Paulo State University) in Botucatu, São Paulo, Brazil. Bouquets were formed with branches of the hybrid *Eucalyptus urophylla* var. *platyphylla*, supported on a 250 ml Erlenmeyer flask with water in a rectangular plastic tray (40 cm long × 35 cm wide × 8 cm high), and were replaced every three to four days, placing the oldest and driest branches next to the new ones to facilitate *Thaumastocoris peregrinus* migration [18]. Adults and third instar nymphs were used in the pathogenicity assay.

Product harvest and Mycoharvester® system

The Mycoharvester® system [Mycoharvester Company, UK] separates pure conidia and agglomerate from fungus colonized substrates using double cyclone technology [19]. A total of 150 g of the substrate + fungus per treatment and replication was placed inside the Mycoharvester® to harvest the conidia. The conidia weight (grams), the number of conidia per gram and the percentage of humidity of the products were obtained to evaluate the harvest quality. In addition, the product obtained (substrate + fungus) was evaluated, both after the fermentation period and following harvesting, to determine the conidia number per gram of dry mass. Twenty-five grams of the substrate were washed with 225 ml Tween 80® [Sigma, Germany] (0.1%). The suspension was shaken for 10 minutes in a KS 15A Orbital Shaker compact agitator [Edmund Bühler GmbH, Germany] at 420 rpm to separate the conidia. The conidia on the substrate were counted before and after passing through the harvesting system. The conidia harvest time was one day. The experiment was repeated independently three times under the same conditions. Fungus production and harvesting experiments were carried out at the Julius Kühn-Institute, Darmstadt, Germany.

Pathogenicity to *Thaumastocoris peregrinus*

The pure conidia with 98.0 ± 1.013% germination rate [20] were suspended in Tween 80® [Sigma, Germany] (0.1%) and their quantity calibrated to concentrations of 1 x 10^6, 10^7, 10^8 and 10^9 of counted conidia/ml to evaluate the pathogenicity, lethal concentration 50 and 90 (LC_{50}, LC_{90}) and lethal time 50 and 90 (LT_{50}, LT_{90}) of the fungus. Then, 2 ml of conidial suspension of each concentration were sprayed using a Potter spray tower [Burkard, UK] (from lowest to highest concentration) on ten *T. peregrinus* adults and ten *T. peregrinus* nymphs (separately), from the laboratory rearing stock for each of the three replications. The control received only Tween 80® (0.1%). The insects were kept in Petri

dishes with their lids covered with voile fabric, allowing gas exchange, and with a *Eucalyptus urophylla* var. *platyphylla* leaf from six-year-old trees to feed the insects. These leaves were placed on a thin layer of hydroretentor gel (1 g/400 ml) [Hydroplan-EB, Empresa de Base & Distribuidora Ltda, Brazil] to maintain their turgescence and prevent the insects from escaping. After applying the fungus on the insects, the Petri dishes were kept in bio-oxygen demand (BOD) incubators at a temperature of 25°C ± 1 and photoperiod of 12h. Each replication (Petri dish) was observed daily for seven days, and dead insects were counted, removed and transferred to humidity chambers to confirm the fungus pathogenicity. The experiment had three replications with 10 nymphs and 10 adults (separately) each, and the tests were repeated twice, totaling 60 insects per insect stage and treatment. The pathogenicity was evaluated at LCBPF (FCA, UNESP).

DNA extraction and PCR

DNA extraction and polymerase chain reactions (PCR) were conducted at the Laboratory of Molecular Biology and Nematology (FCA, UNESP). Mycelium and conidia of IBCB425 isolate with 10 days growth on potato agar dextrose (PDA) culture medium [Kasvi, Italy] at 25°C were used for molecular identification. The microtube containing the sample received 80 µl of 10% Chelex100® resin solution [Bio-Rad Laboratories, USA] and 8 µl of proteinase K (20 mg/ml). Then, the microtube containing the sample was placed in a thermal block at 95°C for 20 minutes [21]. The supernatant was collected for PCR. The PCR reaction for amplification of the ITS1-5.8S-ITS2 region of rDNA was performed in a total volume of 50 µL, using Taq DNA polymerase 1X buffer, 1.5 mM MgCl2, 0.4 µM each of ITS1 (5'-TCCGTAGGTGAACCTGC GG-3') and ITS4 (5'-TCCTCCGCTTATTGATATGC-3') primers, 0.2 mM dNTPs, 0.2 U of Taq DNA polymerase and 25ng of DNA. Amplification was conducted in a thermocycler

[Infinigen, TC-96CG, USA] programmed for initial denaturation at 95°C for 5 min, followed by 30 cycles of 95°C denaturation for 30 sec; annealing at 62°C for 1 min; extension at 72°C for 2 min; and final extension at 72°C for 5 min.

The DNA extraction and PCR reaction (50 μL) products were visualized by 1% agarose gel electrophoresis under UV light and photodocumented. The amplification products were sent to IBTEC Central Laboratory, UNESP/Botucatu, SP, and automated Sanger DNA sequencing was performed (Model: ABI 3500 - Applied Biosystems).

The sequence obtained was used to find the most similar ones deposited in the GenBank, using the BLASTn tool. These sequences were aligned and compared in BLAST and deposited in the GenBank database (http://www.ncbi.nlm.nih.gov). Molecular analysis was important to confirm the fungus species at the beginning and at the end of the research.

Statistical analysis

Generalized linear models with gamma distribution and logarithmic bond function were adjusted for the number of spores per gram before and after harvest, considering a structure of measures repeated by the Generalized Estimation Equation method [22,23,24]. The first considered the conidia mass in grams as a product type factor and the blocks as covariables; the second, water content, considered the product type as factor and the block as covariable; and the third considered the death rate per insect stage as a factor, and time and replication as continuous covariables with block as covariable [22]. The Lethal Concentrations (LC_{50}, LC_{90}) and Lethal Times (LT_{50}, LT_{90}), followed by the confidence interval, were obtained by fitting a logistic regression model with binomial distribution and probit binding function [22]. The procedure used was GENMOD (from

the SAS Statistical Software, SAS University Edition), and the Tukey-Kramer test to compare treatments [25]. The graphs were plotted with SigmaPlot 12.0 software.

Acknowledgments

To the Brazilian institution: "Programa Cooperativo sobre Proteção Florestal (PROTEF) do Instituto de Pesquisas e Estudos Florestais (IPEF)" for financial support and we thank Julius Kühn-Institut (JKI) team. Dr. Phillip John Villani (University of Melbourne, Australia) revised and corrected the English language used in this manuscript.

Author Contributions

Conceived and designed the experiments: SGMV DS CFW. Performed the experiments: SGMV MRV MMD LKB VRC. Analyzed the data: SGMV JRSP. Wrote the paper: SGMV MRV MMD LKB VRC JRSP JCZ DS CFW.

References

1. IBÁ. Anuário estatístico da Indústria Brasileira de Árvores: ano base 2016. Brasília; 2017, 77p. Available from: https://iba.org/images/shared/Biblioteca/IBA_RelatorioAnual2016 _.pdf.
2. Wilcken CF, Soliman EP, Sá LAN, Barbosa LR, Dias TKR, Ferreira-Filho PJ, et al. Bronze bug *Thaumastocoris peregrinus* Carpintero and Dellapé (Hemiptera: Thaumastocoridae) on *Eucalyptus* in Brazil and its distribution. J Plant Prot Res. 2010; 50: 201–206.
3. Wilcken CF, Couto EB, Orlato C, Ferreira-Filho PJ, Firmino DC. Ocorrência do psilídeo-de-concha (*Glycaspis brimblecombei*) em florestas de eucalipto no Brasil. Circular Técnica IPEF. 2003; 201: 1–11.

4. Wilcken CF, Oliveira NC, Sartório RC, Loureiro EB, Bezerra JRN, Rosado-Neto GH. Ocorrência de *Gonipterus scutellatus* Gyllenhal (Coleoptera: Curculionidae) em plantações de eucalipto no estado do Espírito Santo. Arq Inst Biol. 2008; 75: 113–115.
5. Soliman EP, Wilcken CF, Pereira JM, Dias TKR, Zaché B, Dal-Pogetto MHFA, et al. Biology of *Thaumastocoris peregrinus* Carpintero & Dellapé (Hemiptera: Thaumastocoridae) in different eucalyptus species and hybrids. Phytoparasitica. 2012; 40: 223–230.
6. Almeida KEC, Silva JGS, Silva IMA, Costa AL, Laia ML. Ecophysiological analysis of *Eucalyptus camaldulensis* (Dehnh) submitted to attack from *Thaumastocoris peregrinus* (Carpintero & Dellape). Rev Árvore. 2018; 42: e420120.
7. Barbosa LR, Rodrigues AP, Soler LS, Fernandes BV, Castro BMCC, Wilcken CF, et al. Establishment in the field of *Cleruchoides noackae* (Hymenoptera: Mymaridae), an exotic egg parasitoid of *Thaumastocoris peregrinus* (Hemiptera: Thaumastocoridae). Fla Entomol. 2017; 100: 372–374.
8. Souza GK, Pikart TG, Pikart FC, Serrão JE, Wilcken CF, Zanuncio JC. First record of a native heteropteran preying on the introduced eucalyptus pest, *Thaumastocoris peregrinus* (Hemiptera: Thaumastocoridae), in Brazil. Fla Entomol. 2012; 95: 517–520.
9. Dias TKR, Wilcken CF, Soliman EP, Barbosa LR, Serrão JE, Zanuncio JC. Predation of *Thaumastocoris peregrinus* (Hemiptera: Thaumastocoridae) by *Atopozelus opsimus* (Hemiptera: Reduviidae) in Brazil. Isj Invert Surviv J. 2014; 11: 224–227.
10. Dal-Pogetto MHFA, Wilcken CF, Christovam RS, Prado EP, Gimenes MJ Effect of formulated entomopathogenic fungi on red gum lerp psyllid *Glycaspis brimblecombei*. J Forest Res Jpn. 2011; 5: 99–106.

11. Wilcken CF, Oliveira NC. Gorgulho-do-eucalipto *Gonipterus platensis* Marelli. In: Villela EF, Zucchi RA, editors. Pragas Introduzidas no Brasil: Insetos e Ácaros. Piracicaba: Fealq; 2015. pp. 779–791.
12. Lorencetti GAT, Potrich M, Mazaro SM, Lozano ER, Barbosa LR, Menezes MJS, et al. Eficiência de *Beauveria bassiana* Vuill. e *Isaria* sp. para o controle de *Thaumastocoris peregrinus* Carpintero & Dellapé (Hemiptera: Thaumastocoridae). Cienc Florest. 2018; 28: 403–411.
13. Jaronski ST. Mass production on entomopathogenic fungi: state of the art. In: Morales-Ramos JA, Rojas MG, Shapiro-Ilan DI, editors. Mass production of beneficial organisms. Amsterdam: Elsevier Inc; 2014. pp. 357–415.
14. Li Z, Alves SB, Roberts DW, Fan M, Delalibera-Jr I, Tang J, et al. Biological control of insects in Brazil and China: history, current programs and reasons for their successes using entomopathogenic fungi. Biocontrol Sci Techn. 2010; 20: 117–136.
15. Maina UM, Galadima IB, Gambo FM, Zakaria D. A review on the use of entomopathogenic fungi in the management of insect pests of field crops. J Entomol Zool Stud. 2018; 6: 27-32.
16. Liu H, Wang P, Hu Y, Zhao G, Liu H, Li Z, et al. Optimised fermentation conditions and improved collection efficiency using dual cyclone equipment to enhance fungal conidia production. Biocontrol Sci Techn. 2015; 25: 1011–1023.
17. Lüth P, Eiben U. Solid-state fermenter and method for solid-state fermentation United States Prophyta Biologischer Pflanzenschutz GmbH (Malchow, DE). 2003; 6620614. Available from: https://patentimages.storage.googleapis.com/33/5a/c5/332f2e9d923201/US6620614.pdf.

18. Domingues MM, Becchi LK, Velozo SGM, Souza AR, Barbosa LR, Soares MA, et al. Selectivity of mycoinsecticides and a pyrethroid to the egg parasitoid *Cleruchoides noackae* (Hymenoptera: Mymaridae). Sci Rep. 2020; 10: 14617.
19. Bateman R. Operating Instructions for the MycoHarvester Version 5b. 2012. Available from: http://www.dropdata.net/mycoharvester/Operating_Instructions_MycoHarvester_Vb.pdf.
20. Jordan C, Santos PL, Oliveira LRDS, Domingues MM, Gêa BCC, Ribeiro MF, et al. Entomopathogenic fungi as the microbial frontline against the alien *Eucalyptus* pest *Gonipterus platensis* in Brazil. Sci Rep. 2021; 11: 7233.
21. Coombs N, Gough A, Primrose J. Optimisation of DNA and RNA extraction from archival formalin-fixed tissue. Nucleic Acids Res. 1999; 27: 2223-2226.
22. Nelder JA, Wedderburn RW. Generalized linear models. JR Stat Soc Series B. 1972; 135: 370–384.
23. Diggle PJ, Heagerty PJ, Liang KY, Zeger SL. Analysis of Longitudinal Data. 2nd ed; Oxford: Oxford University Press; 2002.
24. Gbur EE, Stroup WW, McCarter KS, Durham S, Young LJ, Christman M, et al. Analysis of Generalized Linear Mixed Models in the Agricultural and Natural Resources Science. American Society of Agronomy, Soil Science Society of America, and Crop Science Society of America, Inc., Wisconsin; 2012.
25. Westfall PH, Tobias RD, Rom D, Wolfinger RD, Hochberg Y. Multiple Comparisons and Multiple Tests Using the SAS System. Carolina do Norte: SAS Institute Inc; 1999.

26. Mochi DA, Monteiro AC, Machado ACR, Yoshida L. Entomopathogenic fungal activity against pupae and adult *Haematobia irritans* (Diptera: Muscidae). Vet Parasitol. 2010; 168: 105–110.
27. Loureiro ES, Batista-Filho A, Almeida JEM, Mendes JM, Pessoa LGA. Eficiência de isolados de *Metarhizium anisopliae* (Metsch.) Sorok. no controle da cigarrinha-da-raiz da cana-de-açúcar, *Mahanarva fimbriolata* (Stal, 1854) (Hemiptera: Cercopidae), em condições de campo. Arq Inst Biol. 2012; 79: 47-53.
28. Mascarin GM, Lopes RB, Delalibera-Jr I, Kamp EFK, Luz C, Faria M. Current status and perspectives of fungal entomopathogens used for microbial control of arthropod pests in Brazil. J Invertebr Pathol. 2018; S0022-2011: 30365-30368.
29. Prakash GVSB, Padmaja V, Kiran RRS. Statistical, optimization of process variables for the large-scale production of *Metarhizium anisopliae* conidiospores in solid-state fermentation. Bioresource Technol. 2008; 99: 1530–1537.
30. Barra-Bucarei L, Vergara P, Cortes A. Conditions to optimize mass production of *Metarhizium anisopliae* (Metschn.) Sorokin 1883 in different substrates. Chil J Agr Res. 2016; 76: 448–454.
31. Van-Breukelen FR, Haemers S, Wijffels RH, Rinzema A. Bioreactor and substrate selection for solid-state cultivation of the malaria mosquito control agent *Metarhizium anisopliae*. Process Biochem. 2011; 46: 751–757.
32. Jaronski ST, Jackson MA. Mass production of entomopathogenic Hypocreales. In: Lacey LA, editor. Manual of techniques in invertebrate pathology. 2nd ed. New York: Academic Press; 2012. Pp. 255-284.
33. Bateman R. The ‘Mycoharvester’: Cleaning up locust control. Int Pest Control. 2003; 45: 76–77.

34. Bateman R. Production Processes for Anamorphic Fungi. IPARC, Silwood Park, Ascot, Berks. 2007. Available from: http://www.dropdata.net/mycoharvester/Mass_production. PDF.

35. Hong TD, Ellis RH, Moore D. Development of a model to predict the effect of temperature and moisture on fungal spore longevity. Ann Bot. 1997; 79: 121-128.

36. Faria M, Hajek AE, Wraight SP. Imbibitional damage in conidia of the entomopathogenic fungi *Beauveria bassiana*, *Metarhizium acridum*, and *Metarhizium anisopliae*. Biol Control. 2009; 51: 346–354.

37. Orduño-Cruz N, Guzmán-Franco AW, Rodríguez-Leyva E, Alatorre-Rosas R, González-Hernández H, Mora-Aguilera G. In vivo selection of entomopathogenic fungal isolates for control of *Diaphorina citri* (Hemiptera: Liviidae). Biol Control. 2015; 90: 1-5.

38. Mascarin GM, Duarte VS, Brandão MM, Delalibera JRI. Natural occurrence of *Zoopththora radicans* (Entomophthorales: Entomophthoraceae) on *Thaumastocoris peregrinus* (Heteroptera: Thaumastocoridae) an invasive pest recently found in Brazil. J Invertebr Pathol. 2012; 110: 401–404.

39. Lorencetti GAT, Potrich M, Mazaro SM, Lozano ER, Gonçalves TE, Dellacort S. Ocorrência espontânea de *Beauveria bassiana* (Bals. Criv.) Vuill. 1912 (Ascomycetes: Clavicipitaceae) sobre *Thaumastocoris peregrinus* (Hemiptera: Thaumastocoridae). Cienc Florest. 2017; 27: 1403–1407.

40. Echeverri-Molina D, Santolamazza-Carbone S. Toxicity of synthetic and biological insecticides against adults of the Eucalyptus snout-beetle *Gonipterus scutellatus* Gyllenhal (Coleoptera: Curculionidae). J Pest Sci. 2010; 83: 297–305.

41. Mascarin GM, Jaronski ST. The production and uses of *Beauveria bassiana* as a microbial insecticide. World J Microbiol Biotechnol. 2016; 32: 177.

42. Ausique JJS, D'Alessandro CP, Conceschi MR, Mascarin GM, Delalibera-Jr I. Efficacy of entomopathogenic fungi against adult *Diaphorina citri* from laboratory to field applications. J Pest Sci. 2017; 90: 947–960.

43. Soliman EP, Castro BMC, Wilcken CF, Firmino AC, Dal-Pogetto MH, Barbosa LR, et al. Susceptibility of *Thaumastocoris peregrinus* (Hemiptera: Thaumastocoridae), a *Eucalyptus* pest, to entomopathogenic fungi. Sci Agr. 2019; 76: 255–260.

44. Abreo E, Simeto S, Corallo B, Martínez G, Lupo S, Altier N. Dual selection of *Beauveria bassiana* strains and complex substrate media for the massive production of submerged propagules with activity against the eucalyptus bronze bug *Thaumastocoris peregrinus*. Biocontrol Sci Techn. 2019; 29: 533-546.

45. Quinelato S, Golo PS, Perinotto WMS, Sá FA, Camargo MG, Angelo IC, et al. Virulence potential of *Metarhizium anisopliae* s.l. isolates on *Rhipicephalus* (*Boophilus*) *microplus* larvae. Vet Parasitol. 2012; 190: 556–565.

46. Loureiro ES, Batista-Filho A, Almeida JEM, Pessoa LGA. Seleção de isolados de *Metarhizium anisopliae* (Metsch.) Sorok. contra a Cigarrinha-da-Raiz da Cana-de-Açúcar *Mahanarva fimbriolata* (Stål) (Hemiptera: Cercopidae) em Laboratório. Neotrop Entomol. 2005; 34: 791-798.

47. Corallo B, Simeto S, Martínez G, Gómez D, Abreo E, Altier N, et al. Entomopathogenic fungi naturally infecting the eucalypt bronze bug, *Thaumastocoris peregrinus* (Heteroptera: Thaumastocoridae), in Uruguay. J Appl Entomol. 2019; 143: 1-14.
