# Supplementary material for: From the dual cyclone harvest performance of single conidium powder to the effect of Metarhizium anisopliae on the management of Thaumastocoris peregrinus (Hemiptera: Thaumastocoridae)
Source: PLoS One. 2023 Mar 27;18(3):e0283543. doi: 10.1371/journal.pone.0283543 (PMC10042367; doi:10.1371/journal.pone.0283543)
Supplement: Attachment — Submitted filename: Response to Reviewers.doc [file pone.0283543.s003.doc]

*Special considerations for this manuscript*:

This manuscript was submitted for its publication in ***PLOS ONE*** (PONE-D-22-33367R1) and we are returning it with the corrections requested by the Subject Editor as a completely revised version.

**Reviewer 1**

*A: We would like to thank you for the critical reading, careful recommendations, and constructive criticism. We have accepted and addressed all suggestions. Our responses to the recommendations are addressed bellow in a point-by-point manner.*

Reviewer #1: Dear editor,

The considerations requested in the first version were fulfilled, however, I added some comments, among them:

"Please review the mortality of control. It is curious that untreated insects show mortality faster than concentrations 10^6 and 10^7. Mortalities higher than 10% in control should have experiments redone".

*A:* *For LT50, when separately comparing the confidence level for 10E6 and control, as well as for 10E7 and control, there is no significant statistic difference (p<0.05) between the means (confidence intervals intersect). The same applies to LT90. As for mortality, the values do not exceed 10%.*

L39, 241, 247, 252, 258, 276 – Third instar nymph

*A:* *We adopted the suggestions provided by R1. The nymphal stage has been specified.*

***OBS:*** *After checking the data, we noticed that the columns in table 1 were mixed up and we corrected them.*

*Reference number 1 has been updated.*
